# Cyclophilin D, Regulator of Mitochondrial Permeability Transition and Bioenergetics, Promotes Adipogenic Differentiation of Mesenchymal Stem Cells

**DOI:** 10.3390/cells15060509

**Published:** 2026-03-13

**Authors:** Chen Yu, Sarah E. Catheline, Roman A. Eliseev

**Affiliations:** 1 Center for Musculoskeletal Research, University of Rochester, Rochester, NY 14642, USA; chen_yu@urmc.rochester.edu (C.Y.); sarah_catheline@urmc.rochester.edu (S.E.C.); 2 Department of Pathology, University of Rochester, Rochester, NY 14642, USA; 3 Department of Pharmacology & Physiology, University of Rochester, Rochester, NY 14642, USA; 4 Wilmot Cancer Institute, University of Rochester, Rochester, NY 14642, USA

**Keywords:** BMSCs, cyclophilin D, mitochondrial permeability transition, adipogenesis, marrow fat

## Abstract

During aging, bone marrow stromal (a.k.a. mesenchymal stem) cells (BMSCs) shift their lineage commitment away from osteogenesis and towards adipogenesis, resulting in bone loss and marrow fat accumulation. We previously reported that during osteogenesis, BMSCs activate mitochondrial oxidative phosphorylation (OXPHOS) at least in part by downregulating cyclophilin D (CypD) expression and, consequently, mitochondrial permeability transition pore (MPTP) activity. We also reported that in contrast, during adipogenesis, BMSCs upregulate CypD and MPTP, activate glycolysis and inhibit OXPHOS. To further study the role of CypD in BMSC bioenergetics, adipogenesis and bone marrow fat accumulation, we used CypD loss-of-function (LOF) or gain-of-function (GOF) models in osteo-adipoprogenitors in vitro and in vivo. We found that CypD LOF and GOF are associated with impaired and enhanced BMSC adipogenesis, respectively, both in vitro and in ectopic bone grafts in vivo. In addition, bioenergetic profiling and metabolomic analyses show evidence of corresponding metabolic reprogramming in CypD LOF and GOF cells. In summary, our study demonstrates the role of CypD-regulated mitochondrial metabolism during BMSC adipogenesis, facilitating the understanding of stem cell fate determination and the molecular mechanism of age-related bone loss as well as bone marrow fat accumulation.

## 1. Introduction

Bone aging is a natural process that results in decreased bone mass and strength. It is associated with an increased risk of fracture and bone disorders such as osteoporosis. Aging induces a series of physiological changes in bone and marrow compartments, resulting in accelerated bone loss, decreased marrow cellularity and increased marrow fat content [1]. While several factors contribute to these changes, the exact underlying mechanisms are not fully understood.

The bone marrow (BM) niche is a complex microenvironment that contains a variety of cell types. It plays a crucial role in regulating the function of hematopoietic stem cells (HSCs) and bone marrow stromal, a.k.a. mesenchymal stem, cells (BMSCs). BMSCs are multipotent cells in the BM niche that can self-renew and differentiate into distinct lineages including osteoblasts, chondrocytes, and adipocytes [2]. Osteolineage cells are essential for bone development, mineralization and remodeling. BMSC-derived osteoblasts are responsible for bone formation since they produce bone matrix proteins. They also secrete a range of growth factors and signaling molecules that can regulate osteogenesis, other marrow cell activities, and even systemic functions [3,4]. Bone marrow adipocytes (BMAs) are the major components of bone marrow adipose tissue (BMAT). They store energy in the form of fat and secrete adipokines, e.g., leptin, adiponectin, and TNFα, to regulate bone cell metabolism [5]. Different from other types of adipose tissue such as white adipose tissue (WAT) and brown adipose tissue (BAT), BMAs have been characterized as a distinct population with unique metabolic features [6].

During aging, BMAT expands and accumulates in the BM niche. At the same time, the homeostasis between bone formation and resorption is lost, which leads to bone loss. Although the mechanisms behind it are not fully understood, several pathological conditions, e.g., estrogen deficiency, mechanical unloading and obesity, have been linked to these age-related changes [7]. It is also widely recognized that aging alters BMSC lineage commitment [8]. During aging, the differentiation potential of BMSCs shifts from osteogenesis toward adipogenesis, resulting in decreased osteolineage cells and increased BMAs.

Mitochondria are critical for stem cell homeostasis and differentiation. While undifferentiated stem cells rely on glycolysis [9], metabolic reprogramming manifested by the activation of oxidative phosphorylation (OXPHOS) is frequently observed during stem cell differentiation. Several studies including ours have shown that OXPHOS activation is required for the osteogenesis of BMSCs [10,11,12,13,14,15]. This is likely in response to the energy demand and high metabolic requirement during osteogenic differentiation and maturation.

The mitochondrial permeability transition pore (MPTP) is a large non-selective calcium-dependent channel in the inner mitochondrial membrane [16,17]. Though the exact structure and formation of the MPTP are still under debate, recent studies indicated that F_O_F_1_-ATP synthase in the inner mitochondrial membrane undergoes conformational changes, forming the MPTP [18]. MPTP opening allows molecules up to 1.5 kDa to diffuse across the membrane. The transient opening of the MPTP is involved in the regulation of calcium homeostasis [19,20], ROS signaling [21,22], stress response [23], and stem cell differentiation [24]. During stem cell pool maintenance, it may play a physiological role by maintaining the prevalence of aerobic glycolysis over OXPHOS. On the other hand, the sustained opening of the MPTP leads to pathological conditions including the dissipation of mitochondrial membrane potential, mitochondrial swelling, and eventual cell death [18]. Cyclophilin D (CypD), encoded by nuclear gene *Ppif*, is a highly conserved peptidyl-prolyl cis–trans isomerase in the mitochondrial matrix. It is the only genetically proven positive regulator of the MPTP that promotes pore opening in response to stimuli such as excessive calcium and ROS. CypD has also been reported to regulate MPTP activity by interacting with p53 [25,26]. Besides its role in MPTP regulation, CypD binds to Bcl-2 to regulate apoptosis [27]. It also affects the assembly of mitochondrial respiratory complexes, respirasomes, and the synthasome [28,29]. CypD also serves as a scaffold for various mitochondrial functional and structural proteins [28]. These various roles of CypD can affect mitochondrial OXPHOS and other functions. Post-translational modifications (PTMs) play an important role in regulating CypD function, including phosphorylation, oxidation, S-nitrosylation, S-glutathionylation, and acetylation. The acetylation of lysine 166 (K166) is critical for CypD pore-opening function, and its deacetylation is catalyzed by mitochondrial deacetylase sirtuin 3 (SIRT3) [30]. Although CypD K166 acetylation’s effect on the isomerase activity of CypD is still unclear, it definitely promotes MPTP opening [28].

CypD and the MPTP strongly influence mitochondrial function and are critical in BMSC fate decisions. We previously reported that during the osteogenesis of BMSCs, CypD is downregulated via the BMP/Smad signaling pathway. This leads to the inhibition of MPTP activity [31] and higher mitochondrial membrane potential and OXPHOS [12,13,14,31,32,33]. We also reported that CypD knockout or pharmacological inhibition by NIM811, a non-immunosuppressive derivative of cyclosporin A and a potent cyclophilin inhibitor, improves bone fracture healing in aging mice [32]. In contrast, we found that the expression of a constitutively active acetylation-mimetic K166Q CypD mutant impairs BMSC osteogenic differentiation and delays fracture healing in adult mice [34]. It also accelerates bone loss in aged mice [33]. Therefore, it appears that mitochondrial dysfunction, a well-known hallmark of aging, concurrent with increased CypD expression in aging [31] may lead to the impaired osteogenic potential of BMSCs. On the other hand, we recently reported that BMAs activate glycolysis and maintain low mitochondrial respiration. This is accompanied by upregulated CypD expression and MPTP activity via C/EBPα- and NF-κB-mediated transcriptional activation of *Ppif* expression [35]. However, it remains unclear whether CypD and the MPTP play a role in adipogenic lineage commitment and age-related BMAT accumulation.

Here, to further explore the role of CypD during the adipogenesis of BMSCs, we used shRNA against a *Ppif* or pCMV6-*caPpif* vector expressing a constitutively active K166Q CypD mutant in the C3H10T1/2 mesenchymal cell line as loss-of-function (LOF) or gain-of-function (GOF) in vitro models, respectively. To study the effects in vivo, we utilized a *Prx1*-Cre mouse to generate the BMSC-specific deletion (*Prx1*-Cre; *Ppif*^f/f^) or overexpression (*Prx1*-Cre; *R26^caPpif/+^*) of CypD. Due to the complexity of the conditions in vivo, we also performed an ectopic bone formation assay using primary mouse BMSCs from the aforementioned mouse models to evaluate the BMSC cell-autonomous effects of CypD manipulation.

## 2. Methods

### 2.1. Cell Culture and Adipogenic Differentiation

C3H10T1/2 cells from ATCC were grown in low-glucose DMEM (Gibco, Waltham, MA, USA) supplied with 10% fetal bovine serum and 1% penicillin–streptomycin. For adipogenic differentiation, cells were induced in DMEM containing 25 mM glucose, 10% fetal bovine serum, 1% penicillin–streptomycin, 1 μM dexamethasone, 0.5 mM IBMX, 1 μM rosiglitazone and 10 μg/mL insulin for 2 days and with rosiglitazone and insulin only afterwards. Media were changed every 2–3 days. Adipogenic differentiation was assessed by Nile Red staining and adipogenic gene expression analysis. Fluorescence intensity was normalized to the cell number assessed via nuclear Hoechst 33342 staining and measured by a Celigo multiwell plate imager (Revvity Inc., Waltham, MA, USA).

### 2.2. Transfection and Stable Clone Selection

As previously described [27], C3H10T1/2 cells were transfected with a SureSilencing CypD shRNA vector (SABiosciences, Frederick, MD, USA) or pCMV6-*caPpif* vector encoding a constitutively active K166Q CypD (caCypD) mutant or corresponding empty vector using X-tremeGENE HP DNA Transfection Reagent (Sigma, St. Louis, MO, USA). For stable CypD knockdown clones, cells were selected with 2 μg/mL puromycin for 2 weeks and maintained in media with puromycin at 0.4 μg/mL. For stable caCypD clones, cells were selected with G418 (Invitrogen, Carlsbad, CA, USA) at 750 μg/mL for 2 weeks and maintained in media with G418 at 200 μg/mL.

### 2.3. Real-Time RT-qPCR

Total RNA was purified using the Qiagen RNeasy kit (Qiagen, Hilden, Germany) and reversed-transcribed into cDNA using the qScript cDNA supermix (Quantabio, Beverly, MA, USA). cDNA was subjected to RT-qPCR. The primer pairs are outlined in Table S3. RT-qPCR was performed with SYBR Green (Quantabio, 95072-012) using the Qiagen RotorGene system. Reaction efficiency was calculated based on a standard curve. β2-microglobulin (*B2m*) was used as a reference gene. The gene expression level was calculated using the 2^−∆∆Ct^ method.

### 2.4. Western Blot

As previously described [35], cells were lysed in buffer containing protease inhibitors and subjected to 4–12% sodium dodecyl sulfate polyacrylamide gel electrophoresis (SDS-PAGE), and polyvinylidene difluoride (PVDF) membrane transfer was performed. The PVDF membrane was blocked in 5% dry milk dissolved in PBST (PBS with 0.1% Tween 20). All antibodies were diluted in either 2% dry milk or 1% BSA in PBST. The membrane was incubated with anti-CypD antibody (RRID: AB 478283, 1:3000), anti-bActin antibody (RRID: AB_476697, 1:30,000) and secondary HRP-conjugated goat anti-mouse or -rabbit antibody (1:3000). Signals were developed with West Femto Substrate (Thermo Fisher Scientific, Waltham, MA, USA). Images were taken using Bio-Rad ChemiDoc Touch Imaging System (Bio-Rad, Hercules, CA, USA).

### 2.5. Calcium Retention Capacity (CRC) Assay

CRC assay was performed as previously described [32]. C3H10T1/2 cells (1 × 10^5^) were permeabilized with 0.01% digitonin for 3 min on ice in a KCI-based buffer [31]. Permeabilization was confirmed with Trypan Blue staining. Then permeabilized cells were washed and resuspended in 100 μL KCI-based buffer containing 1 μM Calcium Green^TM^-5N (Invitrogen, C3737) in a 96-well black-walled and clear-bottom plate. Then cells were exposed to pulses of Ca^2+^ (10 μM increments). The steady-state fluorescent signal after each pulse was measured using a BioTek plate reader (BioTek Instruments, Winooski, VT, USA). Cyclosporin A (CsA) at 1 μM was added to inhibit MPTP opening as a negative control. To calculate CRC, two trendlines were created before and after the Ca^2+^-releasing point. The x-value of the intersection point was calculated as the CRC value.

### 2.6. Seahorse Assay

The oxygen consumption rate (OCR) and extracellular acidification rate (ECAR) were measured using the Seahorse XFe96 Analyzer (Agilent, Santa Clara, CA, USA). C3H10T1/2 cells were plated at a density of 20,000 cells per well in a Seahorse 96-well plate and adipogenically induced for 7 days. Immediately before the experiment, media were replaced with unbuffered DMEM containing 1 mM L-glutamine (Gibco, 25030-08) or 5 mM or 25 mM D-glucose (Sigma, G8270) and no pyruvate (pH 7.4). The OCR and ECAR baseline were measured, and then an inhibitory analysis was performed with sequential injections of 4 μM oligomycin, 2 μM FCCP, 2 μM rotenone + 2 μM antimycin A, and 2 μM 2-deoxyglucose with 3 μg/mL Hoechst 33342. After analysis, the total cell number was measured using a Celigo cytometer (Revvity Inc., Waltham, MA, USA) based on the Hoechst signal. The OXPHOS and glycolytic indexes were calculated as previously described [33].

### 2.7. Metabolomics

Samples were prepared as described previously [33]. Cells were washed with 1× PBS and extracted with 80% methanol. After evaporation under a nitrogen stream, samples were reconstituted in 50% methanol and analyzed using reverse-phase liquid chromatography (LC) with an ion pairing reagent in a Shimadzu HPLC coupled to a Thermo Quantum triple-quad mass spectrometer (MS). The significance level was set as a fold change below or above 1.5 and a *p* value below 0.05. Metabolite enrichment analysis was done using the MetaboAnalyst web server (https://www.metaboanalyst.ca/) accessed on 1 May 2025 and a false discovery rate set below 0.1.

### 2.8. Mouse Strains

C57BL/6J mice were obtained from the Jackson Laboratory (Bar Harbor, ME, USA, RRID: IMSR_JAX:000664) and bred in house. *Ppif*^f/f^ transgenic mice were obtained from the Jackson Laboratory (RRID: IMSR_JAX:005737) and bred in house. *R26^caPpif^* with C57BL/6 genetic background mice were generated by our lab and Dr. George Porter’s lab in the University of Rochester Gene Targeting and Transgenic Core Facility as described previously [33]. In brief, the CypD gain-of-function (GOF) mice contain a knock-in transgene encoding a K166Q constitutively active CypD mutant at the ubiquitously expressed *Rosa26* locus. Constitutive *Prx1*-Cre transgenic mice were obtained from the Jackson Laboratory (RRID: IMSR_JAX:005584) and bred in house. The *Ppif*^f/f^ mice and *R26^caPpif^* mice were crossed to constitutive *Prx1*-Cre transgenic mice, respectively, to generate BMSC-specific CypD deletion (*Prx1*-Cre^+/−^; *Ppif*^f/f^) or overexpression (*Prx1*-Cre^+/−^; *R26^caPpif^*). Animal husbandry and experiments were performed upon the approval of University of Rochester Institutional Animal Care and Use Committee and in accordance with state and federal law. All mice were housed at 23 °C on a 12 h light/dark cycle with free access to water and PicoLab Rodent Diet 20 (LabDiet, St. Louis, MO, USA, #5053). Mice were in group housing when applicable based on weaning. The assessments of animal studies were performed in a blinded and coded manner.

### 2.9. Isolation and Culture of Mouse Primary BMSCs

Primary BMSCs were isolated from tibial and femoral bone marrow. Cells were seeded in physiologically relevant low-glucose DMEM (Gibco, 11885084) supplied with 10% fetal bovine serum and 1% penicillin–streptomycin at 20 × 10^6^ total bone marrow cells per 10 cm dish and incubated at 37 °C, 5% CO_2_ and physiological 5% O_2_. Media were changed every day for three days to remove nonadherent cells. When large colonies were observed on the dish, cells were trypsinized and purified by magnetic immunodepletion with CD45 (Invitrogen, 13045182) and CD31 (Invitrogen, 13031182) antibodies. BMSCs were then seeded and expanded on collagen I -coated dishes. Cells before passage number 10 were used for experiments.

### 2.10. Ectopic Bone Formation Assay

Primary mouse BMSCs were mixed with growth factor-reduced Matrigel (Corning Inc., Corning, NY, USA, CLS354230-1EA) at a concentration of 10^6^ per 50 μL of low-glucose DMEM supplemented with 50 ng/mL of mouse recombinant BMP-2 (R&D systems, Minneapolis, MN, USA, 355-BM-010) and subcutaneously implanted into the backs of immunocompromised nude mice. Bone formation was assessed at 4 weeks after implantation by DEXA scan, μCT analysis, histology, and histomorphometry and IF analysis.

### 2.11. Micro-Computed Tomography (μCT) Analysis

Femurs and tibiae were isolated and cleaned of remaining soft tissue. Afterwards, bones were fixed in 10% neutral buffered formalin for 3 days at 4 °C with gentle agitation. Before scanning, bones were washed with 1× PBS for 30 min at room temperature. Scanning and analysis were performed by Histology, Biochemistry, and Molecular Imaging (HBMI) Core at Center for Musculoskeletal Research (University of Rochester). In brief, bones were scanned using Scanco VivaCT 40 (Scanco Medical AG, Brüttisellen, Switzerland) with high-resolution acquisition (10.5 μm voxel size).

### 2.12. Dual-Energy X-Ray Absorptiometry (DEXA)

Isoflurane-anesthetized mice were scanned, and the area of interest was selected using the Lunar PIXImus 2 system (Piximus, Fitchburg, WI, USA). For our purpose, BMSC grafts were analyzed in terms of bone mineral density (BMD), bone mineral content (BMC) and fat percentage.

### 2.13. Osmium Tetroxide Staining

Bone marrow fat staining was performed as described in the previous literature [36]. Femurs and tibiae were isolated and cleaned to get rid of excessive soft tissue. Then bones were fixed in 10% neutral buffered formalin for 24 h at 4 °C with gentle agitation and then washed with 1× PBS and decalcified in Webb Jee solution (14% EDTA solution) for 2 weeks at 4 °C with gentle agitation. Decalcification solution was replenished every three to four days. Before staining, bones were washed with cool tap water for 1 h. Fresh 1% osmium tetroxide solution was prepared in distilled water with 2.5% potassium dichromate (Sigma). Osmium tetroxide was allowed to dissolve slowly in the solution for 48 h. Bones were incubated in the 1% osmium tetroxide solution for 48 h in a fume hood and then washed with cool tap water for 2 h. μCT scanning and analysis were performed by Histology, Biochemistry, and Molecular Imaging (HBMI) Core at Center for Musculoskeletal Research (University of Rochester). Scanning parameters were set up as described in the literature [36].

### 2.14. Histology

After μCT scanning, bones were decalcified as described above. Paraffin embedding was performed by Histology, Biochemistry, and Molecular Imaging (HBMI) Core at Center for Musculoskeletal Research (University of Rochester). Bones were sectioned at 5 μm and stained for H&E or alcian blue hematoxylin/orange G (ABH/OG) or for IF staining. Slides were imaged using an Olympus VS120 Virtual Slide Microscope (Olympus, Tokyo, Japan). The bone area from ABH/OG sections was quantified using ImageJ software version 1.52t.

### 2.15. Immunofluorescence (IF) Staining

Bone tissue sections were deparaffinized and rehydrated, then air-dried. Antigen retrieval was performed in sodium citrate at 60 °C overnight. Then sections were washed with 1× PBS and blocked with 2.5% goat serum for 30 min and incubated with perilipin-1 primary antibody (Cell Signaling Technology, Danvers, MA, USA, #9349) at 1:100 dilution in PBS + 0.1% Tween-20 and 5% normal goat serum at room temperature for 2 h. Sections were then incubated with anti-rabbit IgG secondary antibody conjugated with Alexa Fluor^®^ 647 (Jackson ImmunoResearch, West Grove, PA, USA, RRID: AB_2338072) at 1:200 dilution for 1 h. Fluoroshield Mounting Medium with DAPI (Abcam, Cambridge, UK, ab104139) was used for counterstaining. The perilipin-positive area and cell number were quantified using ImageJ.

### 2.16. Statistics

A power analysis of in vivo experiment data was performed since it showed the highest variance. It was determined that some quantitative outcomes would require 7 mice per group. At least three independent experiments were performed for each panel of the figures. A two-tailed unpaired *t*-test was used for analysis when two groups were compared, and sample data were normally distributed. When more than two groups were compared, we performed an Ordinary one-way ANOVA with either Tukey’s or Dunnett’s test based on the normal spread of the data. With a significance level at 5%, we calculated the mean values and standard deviations using GraphPad Prism Software version 10.

## 3. Results

### 3.1. CypD Loss of Function Decreases MPTP Activity and Impairs Adipogenesis, Whereas CypD Gain of Function Increases MPTP Activity and Enhances Adipogenesis

**Figure 1:**
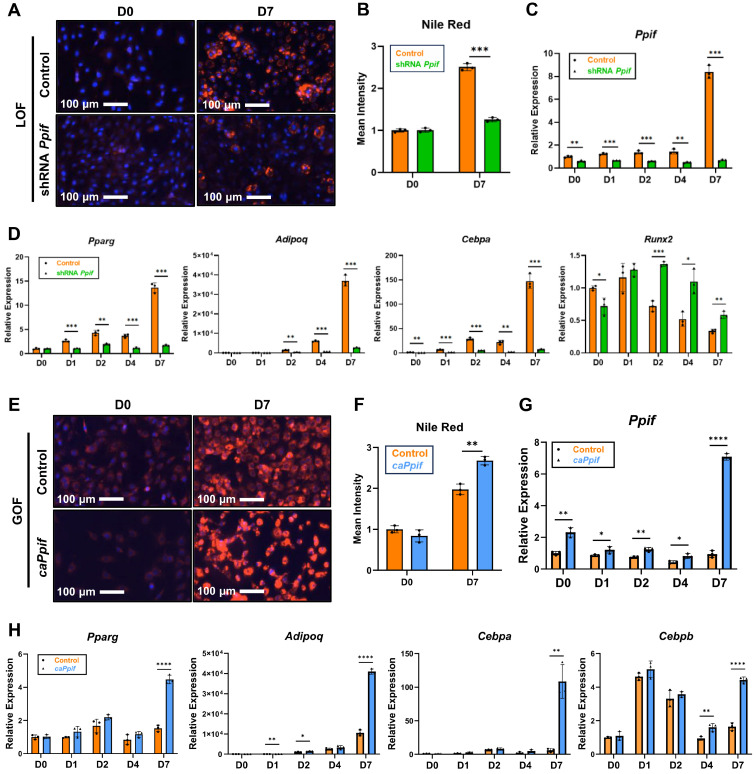
CypD loss of function impairs whereas gain of function enhances adipogenesis on day 7 in C3H10T1/2 cells. C3H10T1/2 cells were transfected with either shRNA against *Ppif* (LOF) or *caPpif* (GOF) vector or control vector. After 48 h, cells were exposed to puromycin or G418 selection for 10 days. Cells were cultured in adipogenic media for 7 days. (**A**,**E**) Cells were stained with Nile Red/Hoechst at D0 and D7. (**B**,**F**) Quantification of Nile Red staining. (**C**,**D**,**G**,**H**) Real-time RT-PCR analysis of *Ppif* and adipogenic gene expression was normalized to *B2m*. Data are mean ± SD (*n* = 3). * *p* < 0.05, ** *p* < 0.01, *** *p* < 0.001, **** *p* < 0.0001 via unpaired *t*-test.

Similar to what is observed in aging humans, C57BL/6J mice show progressively decreased bone mass and strength during aging [37]. Bone marrow fat in aged mice is significantly increased when compared to young mice (Figure S1). In addition, CypD expression is upregulated in aged BMSCs [31,38], and we have previously shown that CypD is increased during adipogenesis [35]. To further study the role of CypD in bone marrow adipogenesis, we generated a stable CypD knockdown (loss-of-function, LOF) cell line using shRNA against the *Ppif* gene as previously described [27]. The mesenchymal cell line C3H10T1/2 is a commonly used model of osteo-adipoprogenitors. These cells were transfected with either shRNA against *Ppif* or control shRNA and stably selected with puromycin. The knockdown of CypD was confirmed by mRNA expression (Figure 1C) and the protein level (Figure S2A). CypD LOF cells showed significantly lower lipid droplet accumulation after 7 days of adipogenesis when compared to the control (Figure 1A,B). The expression of adipogenic genes, *Pparg*, *Adipoq* and *Cebpa*, was significantly decreased in CypD LOF cells vs. controls (Figure 1D), which is consistent with the Nile Red staining. To determine whether CypD knockdown affects osteogenic potential during adipogenesis, we analyzed *Runx2* gene expression, a master regulator of osteogenesis. We found that in CypD LOF cells, *Runx2* was expressed at a higher level and downregulated to a lesser extent during adipogenesis (Figure 1D), suggesting a compromised adipogenic process. NIM811 is a non-immunosuppressive derivative of cyclosporin A and a potent cyclophilin inhibitor. The pharmacological inhibition of CypD by NIM811 also showed decreased lipid droplets (Figure S3A,B) and reduced expression of the adipogenic marker gene *Pparg* in adipo-induced C3H10T1/2 cells on day 7 (Figure S3C). To evaluate MPTP activity, we performed a calcium retention capacity (CRC) assay and found that CypD LOF cells showed significantly higher CRC in undifferentiated cells and adipocytes compared to the control (Figure S2B,C). This result indicates reduced MPTP activity. In summary, these results demonstrate that CypD LOF decreases MPTP activity and is associated with the impaired adipogenesis of osteo-adipoprogenitors, likely in favor of osteogenesis.

As a CypD gain-of-function (GOF) model, we generated a stable *caPpif* overexpression cell line as described in our previous work [27]. We transfected C3H10T1/2 cells with either a pCMV6-*caPpif* vector encoding a constitutively active K166Q CypD (caCypD) mutant or an empty vector control and performed selection with G418. *Ppif* gene expression was higher in CypD GOF cells during adipogenesis when compared to control cells (Figure 1G and Figure S2D). The higher molecular weight of the caCypD protein in the GOF samples is due to the presence of Myc and DDK tags (Figure S2D). Nile Red staining showed that caCypD expression increased lipid droplet accumulation (Figure 1E,F). Adipogenic genes were expressed at a significantly higher level in CypD GOF cells on day 7 of adipogenesis (Figure 1H). CRC assay showed that undifferentiated CypD GOF cells had lower CRC and, therefore, higher MPTP activity when compared to control cells (Figure S2E,F). On day 7 of adipogenesis, CypD GOF cells showed lower CRC when compared to day 0 (Figure S2E,F). However, the CRC of CypD GOF cells and controls was similar on day 7, which might be because they reached the maximum MPTP activity. Taken together, our data demonstrate that CypD GOF via caCypD expression increases MPTP opening and is associated with the enhanced adipogenic potential of osteo-adipoprogenitors.

### 3.2. CypD Loss of Function Improves Whereas Gain of Function Impairs Mitochondrial OXPHOS and Activates Glycolysis

**Figure 2:**
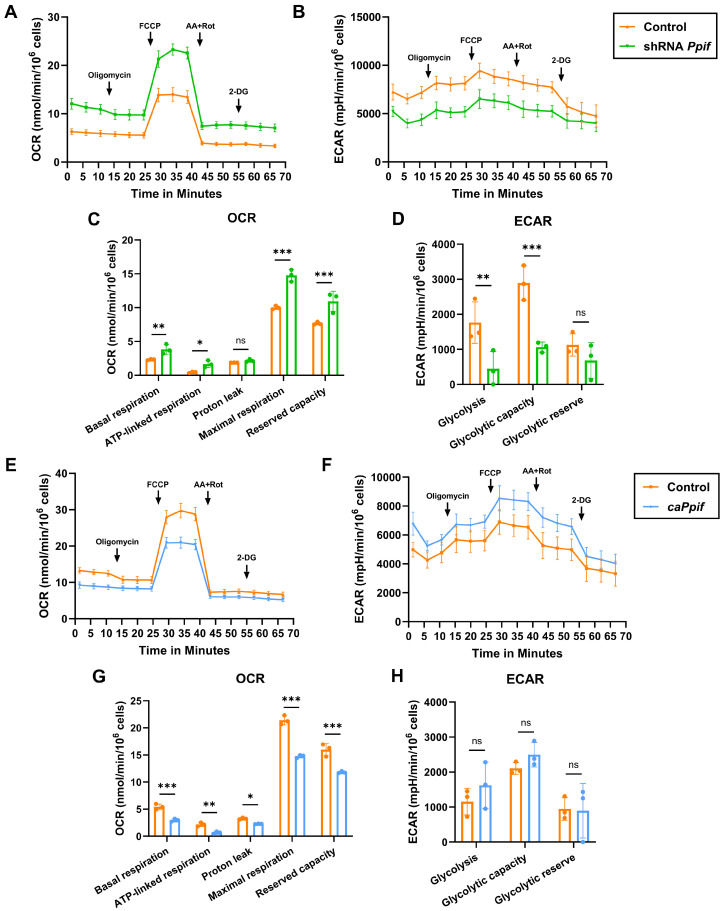
CypD loss of function improves mitochondrial function, whereas CypD gain of function impairs mitochondrial function and activates glycolysis in adipogenically induced C3H10T1/2 cells. CypD LOF and GOF C3H10T1/2 cells were cultured in adipogenic media for 7 days. (**A**,**B**) Kinetic profiles of OCR and ECAR in CypD LOF cells on day 7. (**E**,**F**) Kinetic profiles of OCR and ECAR in CypD GOF cells on day 7. (**C**,**D**,**G**,**H**) Quantification of OCR and ECAR data. Data are mean ± SD (*n* = 3). ^ns^ *p* ≥ 0.05, * *p* < 0.05, ** *p* < 0.01, *** *p* < 0.001 via unpaired *t*-test.

To evaluate the effects of CypD manipulation on cell bioenergetics, we measured OXPHOS and glycolysis levels using a Seahorse XFe96 Analyzer. On day 7 of adipogenesis, CypD knockdown significantly upregulated basal respiration, ATP-linked respiration, maximal respiration, and reserved capacity (Figure 2A,C), indicating that CypD LOF activates mitochondrial OXPHOS function. Furthermore, CypD LOF cells showed significant decreases in basal glycolysis and glycolytic capacity (Figure 2B,D), suggesting a metabolic shift towards OXPHOS in CypD knockdown cells. Collectively, these data demonstrate that CypD LOF decreases MPTP activity, activates mitochondrial OXPHOS function, decreases glycolysis, and impairs the adipogenic potential of osteo-adipoprogenitors, likely in favor of osteogenesis.

In contrast, adipogenically induced CypD GOF cells showed significantly lower basal respiration, ATP-linked respiration, proton leak, maximal respiration, and reserved capacity (Figure 2E,G). However, the glycolytic level remained unchanged when compared to control cells (Figure 2F,H), which might be because adipocytes already activate glycolysis and thus have reached a threshold. In conclusion, these results demonstrate that CypD GOF via caCypD expression increases MPTP opening, decreases OXPHOS, and enhances the adipogenic potential of osteo-adipoprogenitors.

**Figure 3:**
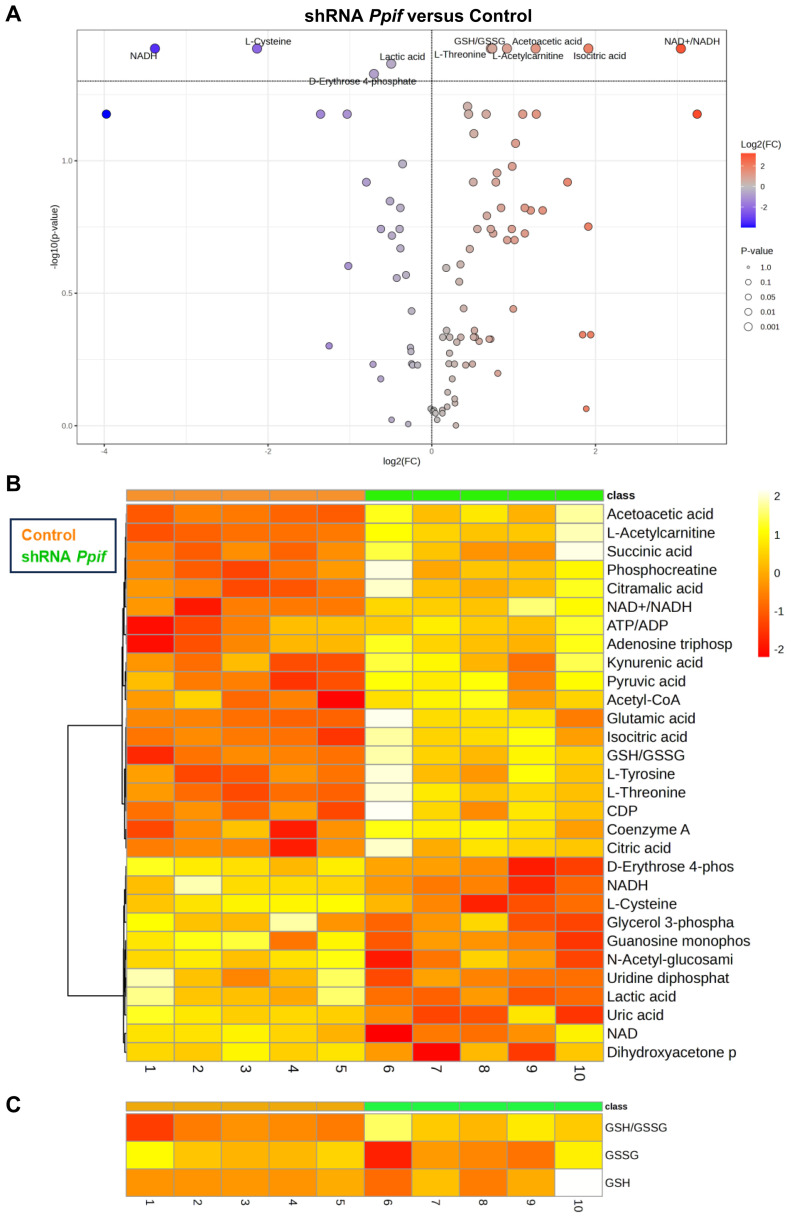
Differential abundance of metabolites was detected in undifferentiated CypD loss-of-function versus control C3H10T1/2 cells. Metabolites were extracted from C3H10T1/2 LOF cells and analyzed by LC-MS. (**A**) Volcano plot showing significantly decreased (in blue) and increased (in red) metabolites. Means and *p* values were calculated using unpaired *t*-test with FDR < 0.1. (**B**) Heatmap showing top 30 differentially expressed metabolites excluding GSH and GSSG. (**C**) Heatmap showing GSH and GSSG levels and GSH/GSSG ratio. Heatmap was based on *p* values with Ward clustering algorithms. Means and *p* values were calculated using unpaired *t*-test.

We next performed untargeted LC-MS metabolomic analysis in undifferentiated CypD LOF and GOF cells. In CypD LOF cells, we found that isocitrate, acetoacetate, L-acetylcarnitine and L-threonine were significantly enriched (Figure 3A). The NAD+/NADH ratio and reduced-to-oxidized glutathione (GSH/GSSG) ratio were also increased, likely suggesting enhanced OXPHOS and lower oxidative stress. Meanwhile, lactate was significantly downregulated, which is consistent with the observed decreased glycolysis level detected via Seahorse profiling (Figure 2B,D). The top 30 differentially expressed metabolites were clustered and visualized by a heatmap (Figure 3B,C). CypD LOF cells showed several upregulated TCA cycle metabolites such as succinate and acetyl-CoA, as well as a higher ATP/ADP ratio (Figure 3B), likely suggesting active mitochondrial function and energy production.

**Figure 4:**
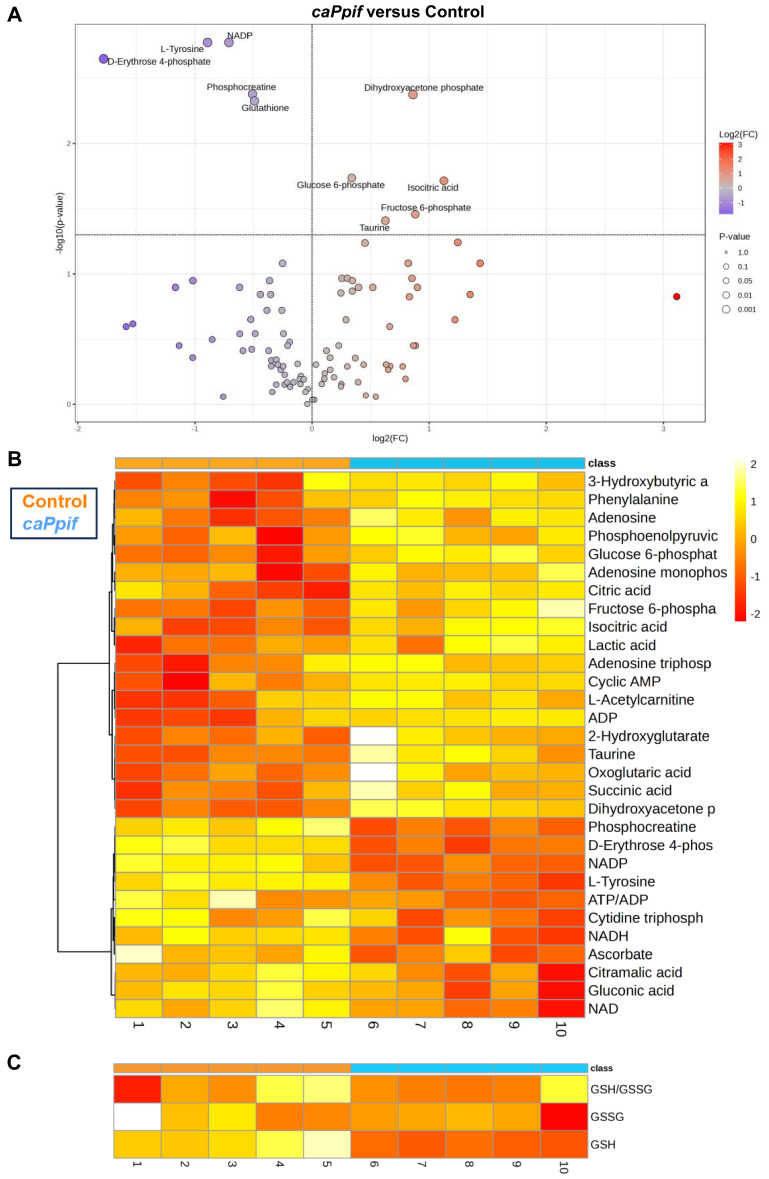
Differential abundance of metabolites was detected in undifferentiated CypD GOF versus control C3H10T1/2 cells. Metabolites were extracted from CypD GOF C3H10T1/2 cells and analyzed by LC-MS. (**A**) Volcano plot showing significantly decreased (in blue) and increased (in red) metabolites. Means and *p* values were calculated using unpaired *t*-test with FDR < 0.1. (**B**) Heatmap showing top 30 differentially expressed metabolites excluding GSH and GSSG. (**C**) Heatmap showing GSH and GSSG expression levels and GSH/GSSG ratio. Heatmap was based on *p* values with Ward clustering algorithms. Means and *p* values were calculated using unpaired *t*-test.

On the other hand, CypD GOF cells significantly increased the levels of glycolytic glucose-6-phosphate, fructose-6-phosphate, and dihydroxyacetone phosphate (DHAP, Figure 4A), consistent with the upregulated glycolysis level detected via Seahorse profiling (Figure 2F,H). Taurine, an amino acid that protects cells against oxidative stress, was enriched in CypD GOF cells, which may indicate compensation against the increased ROS level due to increased MPTP activity. Several metabolites were significantly downregulated including NADP+, L-tyrosine, D-erythrose-4-phosphate, phosphocreatine and glutathione. The hierarchically clustered heatmap also revealed increased glycolysis features such as upregulated lactate (Figure 4B). The decreased ATP/ADP ratio could be attributed to low mitochondrial function and high MPTP activity.

In summary, CypD LOF and GOF induce metabolic reprogramming in mouse osteo-adipoprogenitors. CypD LOF decreases MPTP activity and improves mitochondrial function, whereas CypD GOF increases MPTP activity, impairs mitochondrial function, and activates glycolysis.

### 3.3. BMSC-Specific CypD Gain of Function Increases Fat Accumulation During Ectopic Bone Formation in Mice, While CypD Loss of Function Does the Opposite

**Figure 5:**
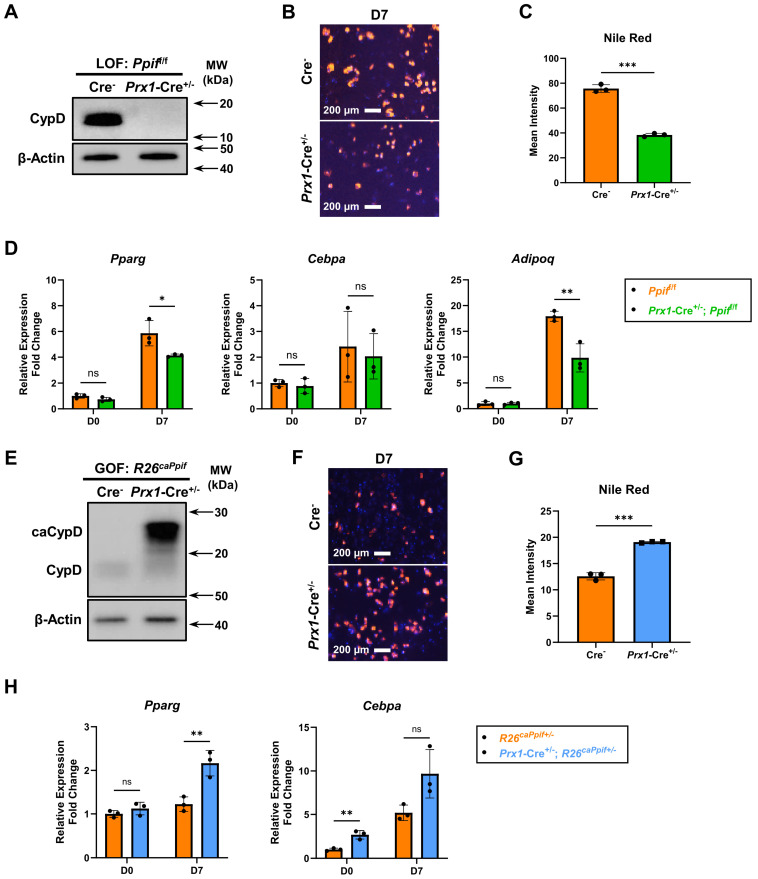
CypD loss of function impairs whereas gain of function enhances adipogenesis in primary mouse BMSCs. Primary BMSCs were isolated from *Prx1*-Cre; *Ppif*^f/f^ (CypD LOF) or *Prx1*-Cre; *R26^caPpif^* (CypD GOF) mice and cultured in adipogenic media for 7 days. (**A**,**E**) Representative Western blot images of CypD protein expression. (**B**,**F**) Cells were stained with Nile Red/Hoechst at D7. (**C**,**G**) Quantification of Nile Red staining. (**D**,**H**) Real-time RT-PCR analysis of adipogenic gene expression was normalized to *B2m*. Data are mean ± SD (*n* = 3). ^ns^ *p* ≥ 0.05, * *p* < 0.05, ** *p* < 0.01, *** *p* < 0.001 via unpaired *t*-test or unpaired multiple *t*-test with Dunn–Bonferroni post hoc test.

To evaluate the effects of CypD LOF in primary mouse BMSCs, we crossed *Prx1*-Cre mice to *Ppif*^f/f^ mice to achieve BMSC-specific CypD deletion. Western blot analysis was performed to validate CypD knockout in BMSCs isolated from *Prx1*-Cre; *Ppif*^f/f^ mice (Figure 5A). After 7-day adipogenic induction, CypD LOF cells showed decreased lipid droplet accumulation when compared to control cells (Figure 5B,C). The expression of adipogenic genes *Pparg*, *Cebpa* and *Adipoq* was also decreased in CypD LOF cells (Figure 5D). Taken together, similarly to C3H10t1/2 cells, CypD deletion in primary mouse BMSCs is associated with impaired adipogenesis.

CypD GOF via caCypD expression in primary mouse BMSCs was achieved by crossing *Prx1*-Cre mice to *R26^caPpif^* mice that we generated earlier and described in our previous reports [34]. The expression of a constitutively active K166Q CypD mutant with Myc and DDK tags was confirmed by Western blot analysis (Figure 5E). Interestingly, in undifferentiated BMSCs, CypD GOF already showed increased lipid droplets compared to control cells (Figure S4A,B). After 7-day adipogenic induction, CypD GOF cells showed significantly higher lipid droplet accumulation (Figure 5F,G). Adipogenic genes *Pparg* and *Cebpa* were expressed at higher levels in CypD GOF BMSCs when compared to control cells (Figure 5H). To summarize, CypD GOF via caCypD expression in primary mouse BMSCs is associated with enhanced adipogenesis.

Next, we sought to evaluate how CypD LOF and GOF in BMSCs affect bone and bone marrow fat accumulation during aging in mice. At 12 months of age, CypD LOF in BMSCs slightly decreased bone marrow fat accumulation when compared to control mice (Figure S5 and Table S1), but the effect was not significant. Unexpectedly, CypD GOF in BMSCs also showed a decrease in femur marrow fat, especially in male mice, where the effect reached significance (Figure S6 and Table S2). In sum, BMSC-specific CypD LOF trended towards a predicted effect of lower bone marrow adiposity, while GOF led to not an increase but a decrease in marrow adiposity. It should be noted that due to the absence of reliable inducible BMSC-specific Cre, we used a constitutive Cre model to manipulate CypD. Therefore, the less pronounced than in vitro effect of CypD LOF in vivo and the opposite to the in vitro trend in CypD GOF in vivo could potentially be due to some systemic compensatory effects [39,40]. More possible explanations are presented in the Section 4.

**Figure 6:**
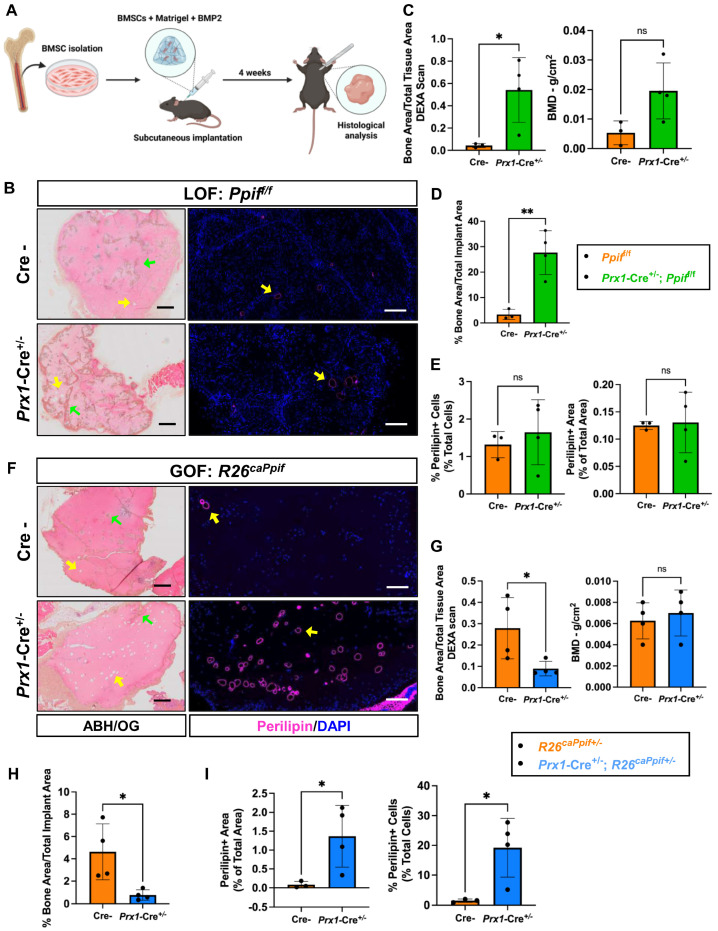
CypD loss of function in primary mouse BMSCs increases ectopic bone formation, whereas CypD gain of function decreases ectopic bone and increases fat formation. Primary BMSCs from *Prx1*-Cre; *Ppif*^f/f^ (CypD LOF) or *Prx1*-Cre; *R26^caPpif^* (CypD GOF) mice were subcutaneously transplanted into nude mice and grafts evaluated after one month. (**A**) Diagram showing procedure of ectopic bone formation assay. Representative 1× alcian blue hematoxylin/orange G staining of ectopic bone implants (left, 500 µM scale bar shown in blue) and 4× perilipin-1 immunofluorescence (IF) staining (right, 200 µM scale bar shown in yellow) from Cre- and *Prx1*-Cre; *Ppif*^f/f^ mice (**B**) or Cre- and *Prx1*-Cre; *R26^caPpif^* mice (**F**). Green arrow indicates ectopic bone. Yellow arrow indicates adipocytes. (**C**,**G**) DEXA scan quantification of bone area/total implant area and bone mineral density in ectopic implants. (**D**,**H**) Bone area/implant area measured in histology images. (**E**,**I**) Perilipin-positive cells as percentage of total cell number and perilipin-positive area as percent of total area measured in histology images. Data are mean ± SD (*n* = 3–4). ^ns^ *p* ≥ 0.05, * *p* < 0.05, ** *p* < 0.01 via unpaired *t*-test.

In order to demonstrate the effects of BMSC-specific CypD manipulation on bone marrow adiposity and minimize systemic interferences, we performed ectopic bone formation assays (Figure 6A). Mouse BMSCs were expanded in vitro, mixed with Matrigel with 50 ng/mL BMP2 and implanted subcutaneously into recipient nude mice. After four weeks, CypD LOF BMSC grafts showed significantly increased ectopic bone area by DEXA scan and ABH/OG staining (Figure 6C,G,H), whereas adipocyte size and number were minimal and similar to Cre- controls (Figure 6C,I). In contrast, CypD GOF BMSC grafts had significantly less ectopic bone (Figure 6B,D,E) and significantly higher adipocyte number and size when compared to Cre- controls (Figure 6B,F). In conclusion, these data confirm that CypD LOF results in the increased osteogenic and reduced adipogenic potential of BMSCs, whereas CypD GOF has the opposite effect.

## 4. Discussion

During adulthood, BMSCs differentiate into either osteoblasts or bone marrow adipocytes, cells that have very different metabolic and biosynthetic requirements [12,13,14]. Previous studies from our group and others show that the activation of mitochondrial OXPHOS is essential for osteogenic differentiation. This activation is achieved at least in part via the downregulation of CypD and resultant suppression of MPTP activity and the possible MPTP-independent effects of CypD [12,13,14,31,32,33,34,35,37]. In contrast, adipocytes upregulate the transcription of the CypD gene, *Ppif*, via the C/EBP-mediated activation of the *Ppif* gene promoter. This leads to increased MPTP opening, less active OXPHOS, and elevated glycolysis. The current study was aimed at further investigating the role of CypD in BMSC fate decisions by manipulating CypD expression and activity during BMSC adipogenesis.

We observed that CypD LOF in the C3H10T1/2 cells and primary BMSCs (*Prx1*-Cre; *Ppif*^f/f^) in vitro inhibited MPTP opening in both undifferentiated cells and adipogenically induced cells. This led to a higher integrity of mitochondrial membranes and stimulation of OXPHOS. During adipogenic induction, CypD LOF BMSCs displayed lower levels of adipogenic factors and higher levels of osteogenic *Runx2*. Runx2 is a major osteogenic factor that is downregulated during adipogenesis. It is also known to suppress adipogenesis [41]. The fact that its expression level remained high in adipogenically induced BMSCs with CypD LOF confirms the role of CypD in BMSC fate decisions. Since our focus here was the effect on adipogenic differentiation, we did not pursue markers of osteogenesis other than Runx2 here. The pro-osteogenic effect of CypD LOF with multiple markers has been demonstrated by us in previous publications [31,32,34,37]. On the other hand, CypD GOF in C3H10T1/2 cells and primary BMSCs (*Prx1*-Cre; *R26^caPpif/+^*) activated the MPTP, reduced OXPHOS activity, and enhanced glycolysis. These metabolic effects of CypD GOF were concurrent with a strong pro-adipogenic effect demonstrated by the higher expression level of adipogenic markers and accumulation of lipids. Notably, even undifferentiated BMSCs with CypD GOF displayed an increased expression of adipogenic *Cebpa* and accumulation of lipid droplets. This indicates an early cell fate shift toward adipogenesis.

Our metabolomic analysis revealed important metabolic changes triggered by CypD manipulation. In undifferentiated CypD LOF cells, key TCA cycle intermediates, including isocitrate, succinate, and acetyl-CoA, were enriched. These changes were accompanied by an increased ATP/ADP ratio, suggesting enhanced OXPHOS. Additionally, the reduction in lactate indicated decreased glycolysis. CypD LOF cells also contained increased levels of acetoacetate, L-threonine, and L-acetylcarnitine. These metabolites are important for maintaining the levels of mitochondrial acetyl-CoA to fuel the TCA cycle and ATP production [42,43,44]. Acetoacetate can also regulate mitochondrial redox balance and protect cells from oxidative stress [45,46,47,48]. L-Cysteine, a precursor for glutathione (GSH), was downregulated, which may suggest active GSH synthesis. Together with the observed increases in the NAD+/NADH and GSH/GSSG ratios, this result supports better protection against oxidative stress. Overall, these metabolic profiling and metabolomic data indicate that CypD LOF activates OXPHOS while lowering MPTP activity and protecting cells from ROS. Conversely, CypD GOF cells showed an accumulation of glycolytic intermediates including glucose-6-phosphate, fructose-6-phosphate, DHAP, and lactate. The ATP/ADP ratio was reduced, which might be due to impaired mitochondrial function. D-erythrose-4-phosphate, an intermediate in the pentose phosphate pathway (PPP), was significantly decreased. This may indicate a less active biosynthesis state for amino acids and nucleotides, comparable to what is observed during aging. In addition, the precursors of fatty acids, isocitrate and citrate, were increased. Together with the fact that 2-hydroxyglutarate (2-HG), a marker of the reductive carboxylation of glutamine, was also increased, this may indicate enhanced fatty acid biosynthesis. The reductive carboxylation of glutamine was shown before to promote lipid biosynthesis [49,50,51]. This is also consistent with our previous findings in the osteoblast-specific CypD GOF model [33]. Collectively, these results indicate that CypD regulates metabolic reprogramming and influences fate decisions in BMSCs.

Our earlier studies showed that global CypD knockout or the use of a systemic inhibitor in mice promotes fracture healing and prevents age-related bone loss [32,37]. Others reported the protective effects of CypD/MPTP LOF against diet-induced obesity [52,53,54,55]. To examine the role of CypD in BMSCs specifically, we used a *Prx1-Cre* mouse model to conditionally knock out CypD. Effects on bone and marrow fat were assessed in these mice. As described in the Section 3, the inhibitory effect of this BMSC-specific LOF of CypD on marrow fat in vivo did not reach significance. Opposite to the in vitro results, the BMSC-specific GOF of CypD in vivo also had an inhibitory effect on marrow fat, which was the most pronounced in male mice. There are several possible explanations for this discrepancy between the in vitro and in vivo results: (1) In the absence of a reliable inducible BMSC-specific Cre model, we used a constitutive model to manipulate CypD, which is prone to compensatory effects. (2) There may be effects of hormones such as growth hormone or estrogen [39,40] and an influence of the microenvironment superseding any cell-autonomous effects. (3) CypD GOF and subsequent higher MPTP activity may induce cell death, leading to a lower number of marrow adipocytes even if adipogenesis is induced by CypD GOF. (4) In this GOF scenario, the fact that male mice had a more pronounced effect may be attributed to a known inhibitory effect of estrogen on the MPTP, leading to less cell death of marrow adipocytes in female mice. All these questions remain unanswered and will be the subject of future studies.

We previously showed that *Col1^CreERt^*-mediated osteoblast-specific CypD LOF protects while GOF impairs osteoblast function and bone homeostasis in aged mice [31,33]. With *Prx1^Cre^*-mediated BMSC-specific CypD LOF and GOF in mice, we did not detect any pronounced effects on bone parameters at 12 months of age. Only one or two of all measured parameters inconsistently showed some significant changes in various experimental groups. This could be attributed to the known limitations of *Prx1*-driven gene manipulation [56] and the above-mentioned constitutive nature of this Cre possibly leading to compensatory effects.

Ectopic bone formation assays provide an alternative strategy to evaluate the cell-autonomous effects of genetic or pharmacological manipulations of BMSCs and assess their multilineage differentiation in vivo [57,58,59]. This assay has its limitations, such as: (1) the absence of mechanical forces affecting bone formation; (2) the lack of bone marrow components, such as immune and hematopoietic cells that can modulate BMSC behavior; (3) the presence of mostly scattered rather than organized bone spicules, etc. Despite these limitations, ectopic bone formation assay remains the assay of choice for uncovering the cell-autonomous effects of BMSCs. Using this assay, we showed that CypD-deficient BMSCs formed significantly more ectopic bone with fewer adipocytes. Conversely, CypD GOF BMSCs showed reduced bone formation and increased fat accumulation. These results are consistent with the in vitro data suggesting a cell-autonomous pro-osteogenic effect of CypD LOF and a pro-adipogenic effect of CypD GOF. Overall, it indicates that CypD is an important regulator of BMSC lineage commitment, likely through its effects on mitochondrial function and MPTP activity.

In summary, here we show that CypD LOF impairs while GOF enhances adipogenesis in BMSCs both in vitro and in ectopic bone in vivo in a cell-autonomous manner. During aging, CypD is upregulated in both BMSCs and osteoblasts [31,38], resulting in higher MPTP activity and mitochondrial dysfunction. The caCypD expression model provides an important tool for understanding bone and marrow aging phenotypes. This model mimics increased CypD/MPTP activity and a metabolic shift toward glycolysis, favoring adipogenesis and leading to age-related accelerated bone loss and accumulated marrow fat.

## Data Availability

The original contributions presented in this study are included in the article/Supplementary Materials. Further inquiries can be directed to the corresponding author.

## Supplementary Materials

The following supporting information can be downloaded at https://www.mdpi.com/article/10.3390/cells15060509/s1, Figure S1: Fat accumulation in C57BL/6J male mice (20 vs. 4 months); Figure S2: CypD knockdown decreases whereas caCypD overexpression increases MPTP opening on day 7 in C3H10T1/2 cells; Figure S3: NIM811, an inhibitor of CypD, impairs adipogenesis in C3H10T1/2 cells; Figure S4: caCypD overexpression in primary mouse BMSCs shows increased lipid droplets at D0; Figure S5: CypD deletion in BMSCs does not affect bone marrow fat in 12-month-old Prx1-Cre;Ppiff/f mice; Figure S6: caCypD overexpression in BMSCs decreases femur marrow fat in 12-month-old male Prx1-Cre;R26caPpif mice; Table S1: Summary of CypD loss-of-function mice at 4 and 12 months old; Table S2: Summary of CypD gain-of-function mice at 4 and 12 months old; Table S3: Primer sequences used for real-time RT-PCR.

## Abbreviations

BMSCs	bone marrow stromal cells
HSCs	hematopoietic stem cells
BMAs	bone marrow adipocytes
OBs	osteoblasts
BM	bone marrow
BMAT	bone marrow adipose tissue
WAT	white adipose tissue
BAT	brown adipose tissue
OXPHOS	oxidative phosphorylation
MPTP	mitochondrial permeability transition pore
CypD	cyclophilin D
caCypD	K166Q constitutively active CypD
ROS	reactive oxygen species
PTMs	post-translational modifications
LOF	loss of function
GOF	gain of function
OCR	oxygen consumption rate
ECAR	extracellular acidification rate
LC-MS	liquid chromatography–mass spectrometry
BMD	bone mineral density
BMC	bone mineral content
ABH/OG	alcian blue hematoxylin/orange G
CRC	calcium retention capacity
DHAP	dihydroxyacetone phosphate
GSH	glutathione
GSSG	glutathione disulfide
PPP	pentose phosphate pathway
2-HG	2-hydroxyglutarate
