# Supplementary material for: Cyclophilin D, Regulator of Mitochondrial Permeability Transition and Bioenergetics, Promotes Adipogenic Differentiation of Mesenchymal Stem Cells
Source: Cells. 2026 Mar 13;15(6):509. doi: 10.3390/cells15060509 (PMC13025710; doi:10.3390/cells15060509)
Supplement: Supplementary file 1 [file cells-15-00509-s001.zip › cells-4127823-supplementary figures,tables.docx]

**Figure S1 Fat accumulation in C57BL/6J male mice (20 vs 4 months).** Femur and tibia were collected and fixed as described previously. Marrow fat was measured by quantitative volumetric μCT analysis after osmium tetroxide staining. Adipose tissue volume (AV) was normalized to total volume (TV). Data are mean±SD (n=4). *p<0.05, **p<0.01, ***p<0.001 via unpaired *t*-test.





**Figure S2 CypD knockdown decreases whereas caCypD overexpression increases MPTP opening at day 7 in C3H10T1/2 cells.** C3H10T1/2 cells were transfected and cultured as described above. A) and D) Representative western blot images of CypD or caCypD protein expression. B) and E) CRC assay measures calcium uptake by mitochondria at D0 and D7. C) and F) CRC was calculated and plotted as fold change of D7 control. Data are mean±SD (n=3 or n=4). *p<0.05, **p<0.01, ***p<0.001 via unpaired *t*-test.





**Figure S3 NIM811, an inhibitor of CypD, impairs adipogenesis in C3H10T1/2 cells.** C3H10T1/2 cells were cultured in adipogenic media with or without NIM811 for 7 days. A) Cells were stained with Nile Red/Hoechst at D14. B) Quantification of Nile Red staining. C) Real-time RT-PCR analysis of adipogenic gene *Pparg* were normalized to *B2m*. Data are mean±SD (n=3). *p<0.05, **p<0.01, ***p<0.001 via unpaired *t*-test.





**Figure S4 caCypD overexpression in primary mouse BMSCs shows increased lipid droplets at D0.** Primary BMSCs were isolated from *Prx1*-Cre;*R26^caPpif^* mice and cultured in DMEM media. A) Undifferentiated cells were stained with Nile Red/Hoechst. B) Quantification of Nile Red staining. Data are mean±SD (n=6). *p<0.05, **p<0.01, ***p<0.001 via unpaired *t*-test.





**Figure S5 CypD deletion in BMSCs does not affect bone marrow fat in 12-month-old *Prx1*-Cre;*Ppif*^f/f^ mice.** Femur and tibia were collected from 12-month-old *Prx1*-Cre;*Ppif*^f/f^ mice. Marrow fat was measured by quantitative volumetric μCT analysis after osmium tetroxide staining. A) Representative H&E staining images of proximal femur and distal tibia. B) Representative Perilipin-1 IF staining images of distal tibia. C) female and E) male marrow fat volume was normalized to total volume. Representative μCT marrow fat images of femur and tibia from D) female and F) male mice. AV/TV = adipose tissue fraction volume. Data are mean±SD (n=6-9). *p<0.05, **p<0.01, ***p<0.001 via unpaired *t*-test.





**Figure S6 caCypD overexpression in BMSCs decreases femur marrow fat in 12-month-old male *Prx1*-Cre;*R26^caPpif^* mice.** Femur and tibia were collected from 12-month-old *Prx1*-Cre;*R26^caPpif^* mice. Marrow fat was measured by quantitative volumetric μCT analysis after osmium tetroxide staining. A) Representative H&E staining images of proximal femur and distal tibia. B) Representative Perilipin-1 IF staining images of distal tibia. C) female and E) male marrow fat volume were normalized to total volume. Representative μCT marrow fat images of femur and tibia from D) female and F) male mice. AV/TV = adipose tissue fraction volume. Data are mean±SD (n=7-9). *p<0.05, **p<0.01, ***p<0.001 via unpaired *t*-test.

**Table S1 Summary of CypD loss-of-function mice at 4 and 12 months old.**

Data are mean±SD (n=7-9). *p<0.05 via unpaired *t*-test.





**Table S2 Summary of CypD gain-of-function mice at 4 and 12 months old.**

Data are mean±SD (n=7-9). *p<0.05 via unpaired *t*-test.
