# Supplementary material for: Cyclophilin D, Regulator of Mitochondrial Permeability Transition and Bioenergetics, Promotes Adipogenic Differentiation of Mesenchymal Stem Cells
Source: Cells. 2026 Mar 13;15(6):509. doi: 10.3390/cells15060509 (PMC13025710; doi:10.3390/cells15060509)
Supplement: Supplementary file 1 [file cells-15-00509-s001.zip › cells-4127823-supplementary table S3.pdf]

Table S3

Primer	Sequence 5’-3’
Mouse *B2m*_forward	AATGGGAAGCCGAACATAC
Mouse *B2m*_reverse	CCATACTGGCATGCTTAACT
Mouse *Ppif*_forward	CATGTACCC GAACAGAAC
Mouse *Ppif*_reverse	CATGTACCC GAACAGAAC
Mouse *Adipoq*_forward	GAGAAGGGAGAGAAAGGAGATG
Mouse *Adipoq*_reverse	TGAGCGATACACATAAGCGG
Mouse *Cebpa*_forward	ATAAGAACAGCAACGAGTACC
Mouse *Cebpa*_reverse	GCGGTCATTGTCACTGGTC
Mouse *Pparg*_forward	GGCCTCCCTGATGAATAAAG
Mouse *Pparg*_reverse	GCCAAGTCACTGTCATCTAAT
Mouse *Cebpb*_forward	CCTTAGACCCATGGAAGTGG
Mouse *Cebpb*_reverse	GGGCTGAAGTCGATGGC
Mouse *Runx2*_forward	CCGGGAATGATGAGAACTAC
Mouse *Runx2*_reverse	CCGTCCACTGTCACTTTAATA

Table S3. Primer sequences used for real-time RT-PCR.
